# Prospective Validation of an *Ex Vivo*, Patient-Derived 3D Spheroid Model for Response Predictions in Newly Diagnosed Ovarian Cancer

**DOI:** 10.1038/s41598-019-47578-7

**Published:** 2019-08-01

**Authors:** Stephen Shuford, Christine Wilhelm, Melissa Rayner, Ashley Elrod, Melissa Millard, Christina Mattingly, Alina Lotstein, Ashley M. Smith, Qi Jin Guo, Lauren O’Donnell, Jeffrey Elder, Larry Puls, S. John Weroha, Xiaonan Hou, Valentina Zanfagnin, Alpa Nick, Michael P. Stany, G. Larry Maxwell, Thomas Conrads, Anil K. Sood, David Orr, Lillia M. Holmes, Matthew Gevaert, Howland E. Crosswell, Teresa M. DesRochers

**Affiliations:** 1 KIYATEC, Inc., Greenville, South Carolina USA; 2 Prisma Health, Greenville, South Carolina USA; 3 Department of Medical Oncology, Mayo Clinic, Rochester, Minnesota USA; 4 Saint Thomas Health/Ascension, Nashville, Tennessee USA; 5 Department of Obstetrics and Gynecology, Inova Fairfax Medical Campus, Falls Church, Virginia USA; 6 Departments of Gynecologic Oncology and Cancer Biology, MD Anderson Cancer Center, Houston, Texas USA; 7 Present Address: USC School of Medicine, Greenville, South Carolina USA; 8 Present Address: SYNNEX Corporation, Greenville, South Carolina USA; 9 Present Address: Tennessee Oncology, Nashville, Tennessee USA; 10 Present Address: MacuLogix, Inc, Middletown, Pennsylvania USA; 11 Present Address: Bon Secours St. Francis, Greenville, South Carolina USA; 12 Present Address: MUSC College of Medicine, Charleston, South Carolina USA; 13 Present Address: ECPI University, Greenville, South Carolina USA

**Keywords:** Ovarian cancer, Tumour biomarkers, Cancer models

## Abstract

Although 70–80% of newly diagnosed ovarian cancer patients respond to first-line therapy, almost all relapse and five-year survival remains below 50%. One strategy to increase five-year survival is prolonging time to relapse by improving first-line therapy response. However, no biomarker today can accurately predict individual response to therapy. In this study, we present analytical and prospective clinical validation of a new test that utilizes primary patient tissue in 3D cell culture to make patient-specific response predictions prior to initiation of treatment in the clinic. Test results were generated within seven days of tissue receipt from newly diagnosed ovarian cancer patients obtained at standard surgical debulking or laparoscopic biopsy. Patients were followed for clinical response to chemotherapy. In a study population of 44, the 32 test-predicted Responders had a clinical response rate of 100% across both adjuvant and neoadjuvant treated populations with an overall prediction accuracy of 89% (39 of 44, p < 0.0001). The test also functioned as a prognostic readout with test-predicted Responders having a significantly increased progression-free survival compared to test-predicted Non-Responders, p = 0.01. This correlative accuracy establishes the test’s potential to benefit ovarian cancer patients through accurate prediction of patient-specific response before treatment.

## Introduction

Although the current first-line treatment paradigm for newly diagnosed ovarian cancer patients is initially effective in 70–80% of cases, most patients will relapse^1^ and fewer than half of all women diagnosed with ovarian cancer will survive beyond five years^2^. Overall survival may be prolonged by lengthening the time to initial progression as this time interval has an impact upon clinical treatment and response^3–5^. There are no biomarkers currently available to accurately predict patient response to first- or second-line therapy, hampering efforts to increase overall survival by this strategy. A test that could make an accurate response prediction on a case-by-case basis at diagnosis could benefit the 20–30% of patients that do not respond initially and improve their clinical outcomes.

The treatment paradigm for newly diagnosed ovarian cancer patients is well defined by clinical guidelines set by the National Comprehensive Cancer Network (NCCN) and usually follows one of two paths, either surgical debulking followed by six cycles of platinum/paclitaxel chemotherapy or laparoscopic biopsy followed by 3–4 cycles of platinum/paclitaxel chemotherapy, interval debulking, and an additional 3 cycles of the same chemotherapy^6–8^. Upon relapse patients are classified as “platinum sensitive” or “platinum resistant” dependent upon the length of time to recurrence following the completion of first-line, platinum-based chemotherapy; “platinum sensitive” patients maintain disease-free status greater than six months while “platinum resistant” patients progress in less than 6 months^9,10^.

Currently, tests to support clinical treatment decisions in oncology generally fall into one of two categories, neither of which identifies individual Responder or Non-Responder patients. In one category, tests use the presence of single biomarkers or mutations (e.g., BRCA, HER2, EGFR, PD-1, BRAF, etc.) to establish patients that harbor actionable markers and therefore may respond to targeted therapies. Many tested patients have no actionable markers^11^; and many additional patients who are treated based on positive mutation or biomarker status do not respond to the marker-associated treatment^12–14^. In the second category, multiple gene mutations in defined sets place tested patients within a defined prognostic group of clinical outcomes based on a quantitative, individualized value^15–18^. By this determined value, patients are accurately identified as more or less likely to benefit, however, the tests do not predict individual response, and there are still many patients who receive treatment without a response. The clinical accuracy for both categories of test is sometimes limited because results are not a consequence of patient-specific tissue interaction with drug, but rather are generated in the complete absence of cell-drug interaction. Infectious disease treatment paradigms have solved this limit to clinical utility by obtaining patient-specific evidence of response through direct exposure of live patient-specific bacteria to various drugs and routinely and effectively use *in vitro* response to guide regimen selection. If comparably effective, an analogous process in cancer using live patient-specific cancer cells to effectively select patient-specific cancer therapies would have tremendous beneficial impact on clinical care in oncology.

Throughout the past few decades many *in vitro* chemotherapy resistance and sensitivity assays (CRSAs) have been developed using various techniques including 2D cell culture, tissue slices, and soft agar assays^19–23^. None of these previously developed assays are recommended by the American Society of Clinical Oncology for patient treatment selection outside of clinical trials and there is debate within the oncology community as to their utility^24^. Stated reasons for the current lack of adoption include insufficient evidence of clinical benefit to patients and lack of demonstrated clinical utility to physicians. The predictive accuracy of CRSAs may be a function of the *in vitro* techniques utilized. For example, 2D cell culture has low success rates with primary cells and has limited biologic fidelity and thus limited sensitivity for patient-specific drug response^25,26^. To address these shortcomings, we have developed an *ex vivo* 3D cell culture drug response test with a rapid turnaround time and a validated, scalable process. The test combines the increased cell culture success rate and biological fidelity of 3D cell culture with standardized procedures resulting in increased replicate testing, decreased intra-assay heterogeneity, and the testing of standard of care agents utilized in the treatment of ovarian cancer.

Here, we provide the analytical validation and initial prospective clinical validation of this test to accurately predict patient-specific response to first-line chemotherapy in newly diagnosed ovarian cancer patients prior to treatment initiation. The response prediction encompasses all drugs received by the patients as part of their first-line and/or second-line therapy, enabling patient matched correlation between test result and patient clinical response to standard of care therapy. This correlation provides robust evidence of the future potential use of this test in a clinical setting to increase positive outcomes and benefit patients through better informed treatment decision making.

## Results

### Analytical validation of the test

**Figure 1:**
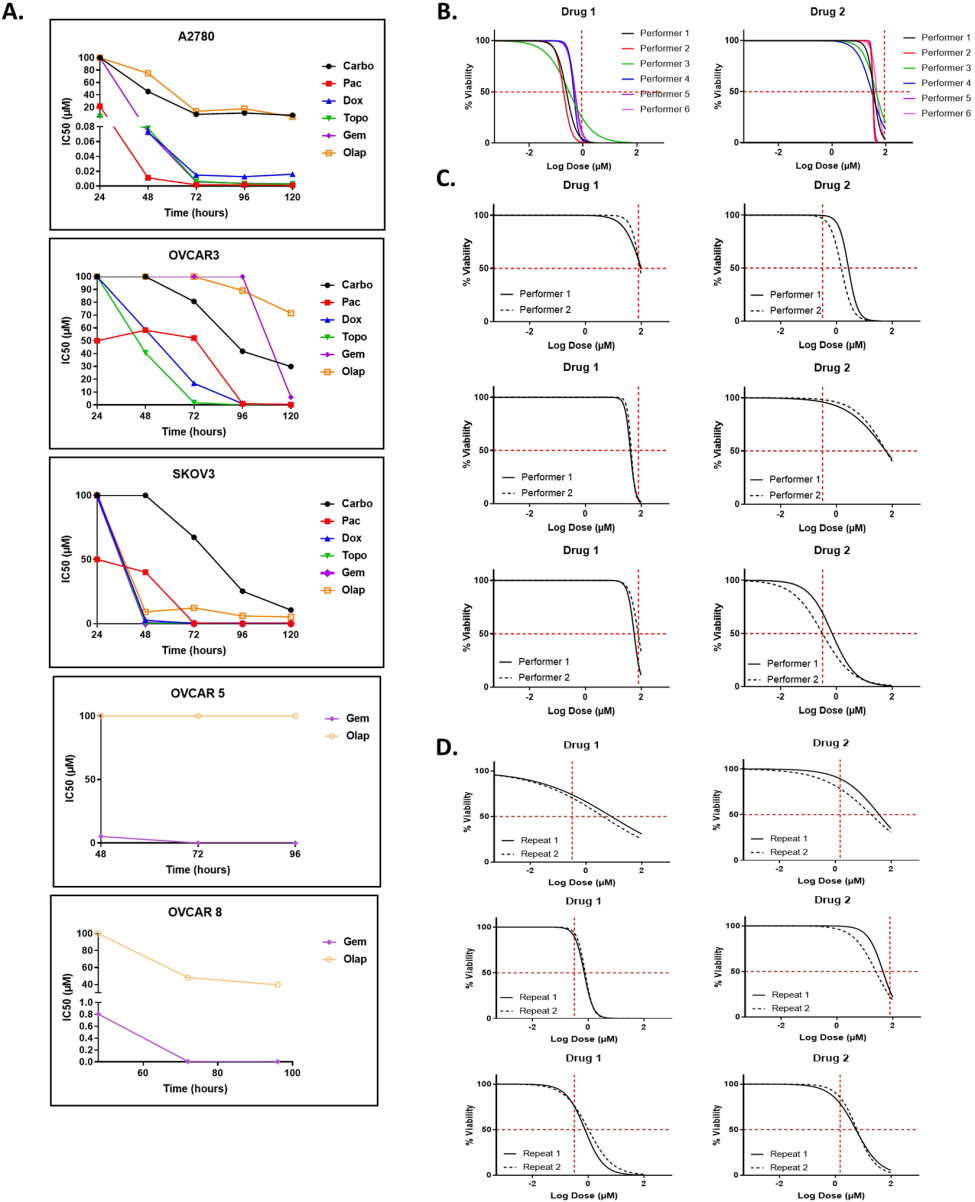
Analytical validation of the assay. (**A**) Timing analysis of optimal drug response in five ovarian cancer cell lines. Abbreviations: Carbo = carboplatin, Pac = paclitaxel, Dox = doxorubicin, Topo = topotecan, Gem = gemcitabine, Olap = olaparib. (**B**–**D**) Representative data generated testing the intra- and inter-assay reproducibility. Reproducibility was defined as having similar drug response readouts which are indicated by the vertical red hash lines in the graphs. (**B**) Overlapping non-linear regression curves of drug response in the assay performed by 6 separate operators on one primary tissue sample. Each color represents a curve generated by a different operator. (**C**) Repetition of the assay by two individual operators for 3 different cell lines and 2 drugs each. (**D**) Repetition of the assay by a single operator for 3 separate primary tissue samples and 2 drugs each. At each data point, n = 7.

In order to produce a test that could perform universally across the heterogeneity of patient samples, we first analytically validated the test using a combination of cell lines and primary patient samples (Fig. 1). The timing of drug incubation was standardized using cell lines due to the limited cell numbers reproducibly available from any one primary tumor and the need to produce reliable, large scale results for this aspect of the test (Fig. 1A). If, upon individual primary sample testing, it had become obvious that no drug response was found in 20 primary samples, we would have re-evaluated both the drug curve and the timing of exposure. Drug response was interrogated from 24 to 120 hours following addition to 3D spheroids and IC50s were calculated and graphed over time. A single, optimal time period of 72 hours was established that allowed the differentiation of test response/non-response without falling too close to either extremes of the dose response curves.

Inter-assay reproducibility was performed on one primary sample (Fig. 1B) and three cell lines (Fig. 1C) following the standard SOPs for the response prediction test. The original sample, primary tumor or cell line, was initially processed to single cells in suspension by one performer and then separated among all performers equally who then performed individual spheroid formation, drug dosing, viability assays, and data analysis. The similarity of results was judged against Response/Non-Response categories for each drug (red hash lines). For the primary sample, six separate performers at KIYATEC were able to produce similar results within the range of drug response categories. Similar results were seen when the test was performed on cell lines between two different performers with a mix of Response and Non-Response to the tested drugs that were replicated between performers. Intra-assay reproducibility was performed by one person on three different primary samples with successful reproduction of their own results on separate occasions (Fig. 1D).

### Characteristics of the patients

**Figure 2:**
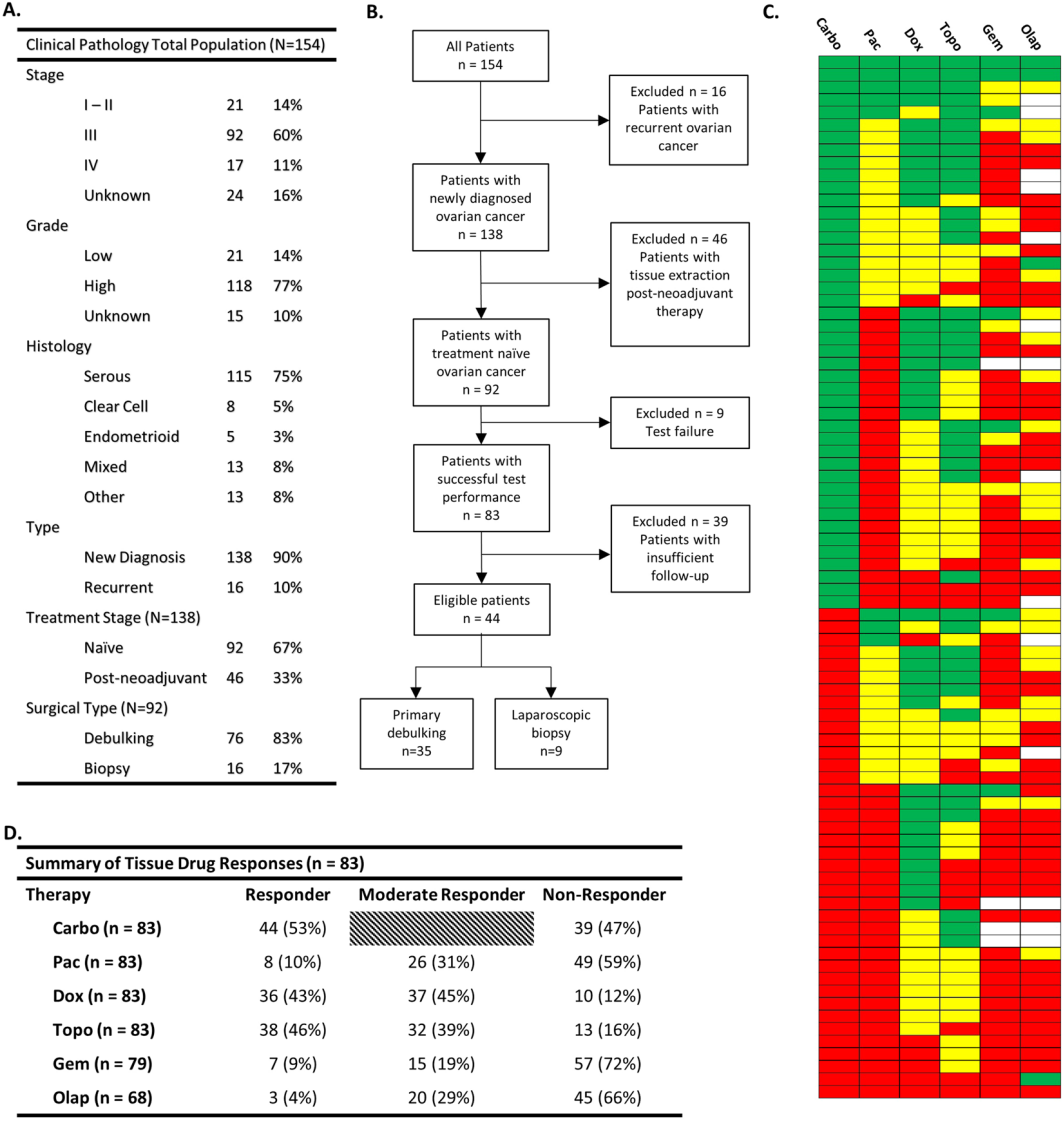
Descriptive characteristics of the patients enrolled in the prospective clinical validation study. (**A**) Clinical pathology of all patients enrolled in the study. (**B**) Flow chart of the patient inclusion process. (**C**) Drug response category for all 83 successfully tested patient samples. Green indicates Responder, yellow Moderate Responder, and red Non-Responder. Blank squares indicate that drug was not tested. (**D**) Summary table of responses indicated in C. Carboplatin has only two categories, Responder and Non-Responder. Abbreviations: Carbo = carboplatin, Pac = paclitaxel, Dox = doxorubicin, Topo = topotecan, Gem = gemcitabine, Olap = olaparib.

A total of 154 women with a diagnosis of ovarian cancer were enrolled from November 2014 to November 2018. Ninety-two were eligible for this analysis with newly diagnosed, treatment naive ovarian cancer, receipt of fresh tissue from either a primary debulking surgery or laparoscopic biopsy, 76 and 16 respectively (Fig. 2), and receipt of platinum-based chemotherapy. Eighty-three tissues (90%) were successfully tested for drug response to at least carboplatin and paclitaxel. Eight of the nine test failures were due to poor viability of the cells isolated from the samples either at the initial tissue digestion or in the untreated controls over the course of the assay. Only one sample failed due to technician error. All 83 successfully assayed tissues were tested for categorical response to carboplatin, paclitaxel, and cell numbers permitting, approved second- and third-line agents, including doxorubicin, topotecan, gemcitabine, and olaparib (Fig. 2C,D). Every sample except one had a measurable level of response or moderate response to at least one agent. The one patient sample with no response had no discernably obvious reasons for the lack of drug response in the assay. Multiple patient samples had a response to both carboplatin and paclitaxel (20 of 83, 24%) while (38 of 83, 46%) responded to carboplatin or paclitaxel alone with the majority of those (24 of 38, 63%) being a response to carboplatin rather than paclitaxel. Importantly, 30% of patients (25 of 83) did not respond to either carboplatin or paclitaxel.

**Figure 3:**
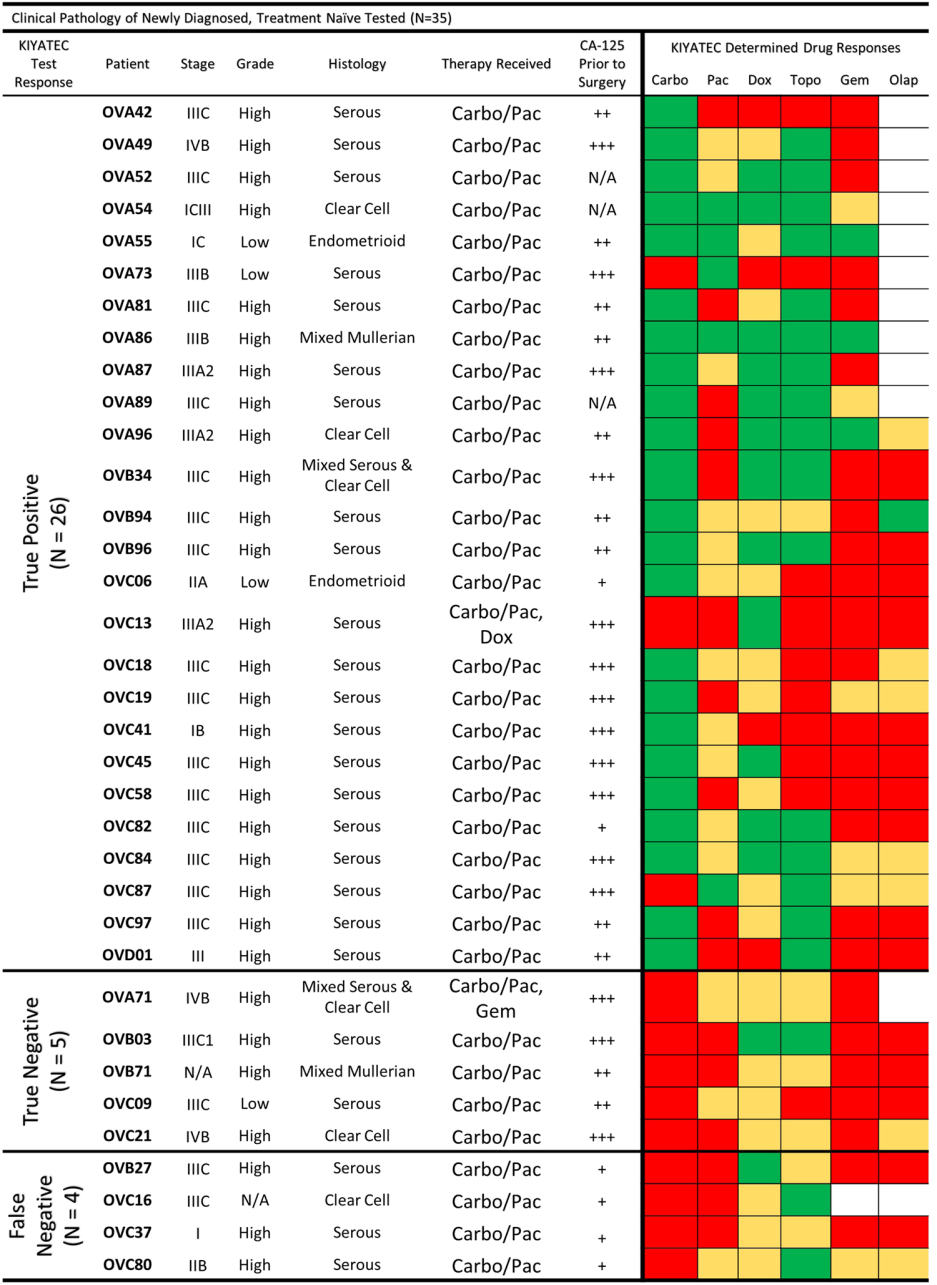
Clinical pathology of all patients in the newly diagnosed, treatment naïve, primary debulking category. Green indicates a Test Responder, Yellow a Test Moderate Responder, and Red a Test Non-Responder. Abbreviations: Carbo = carboplatin, Pac = paclitaxel, Dox = doxorubicin, Topo = topotecan, Gem = gemcitabine, Olap = olaparib; + = CA-125 ≤ 60, ++ = CA-125 > 60, <500, +++ = CA-125 ≥ 500.

Of the 76 primary debulking patients, 67 (88%) had successful tests performed on their excised tumor tissue. Thirty-five of these patients had the required minimum six months of clinical follow-up to determine clinical response to standard first-line combination carboplatin/paclitaxel therapy (Fig. 3). These patients were classified as clinical responders or non-responders based on their disease status at six months following the completion of chemotherapy. No stratification was made based on achieving complete cytoreduction (R0)^27^, stage, grade, or distribution of disease^28^. The 35 patients with sufficient clinical follow-up for test correlation encompassed multiple stages, grades, and histological subtypes. All 16 of the laparoscopic biopsy patients (100%) had successful tests performed on their excised tumor tissue and nine patients had CT imaging data prior to biopsy and directly prior to interval debulking surgery to categorize their response to neoadjuvant chemotherapy^29,30^.

### Test response predicts clinical response to first-line adjuvant chemotherapy in newly diagnosed ovarian cancer with primary debulking

**Figure 4:**
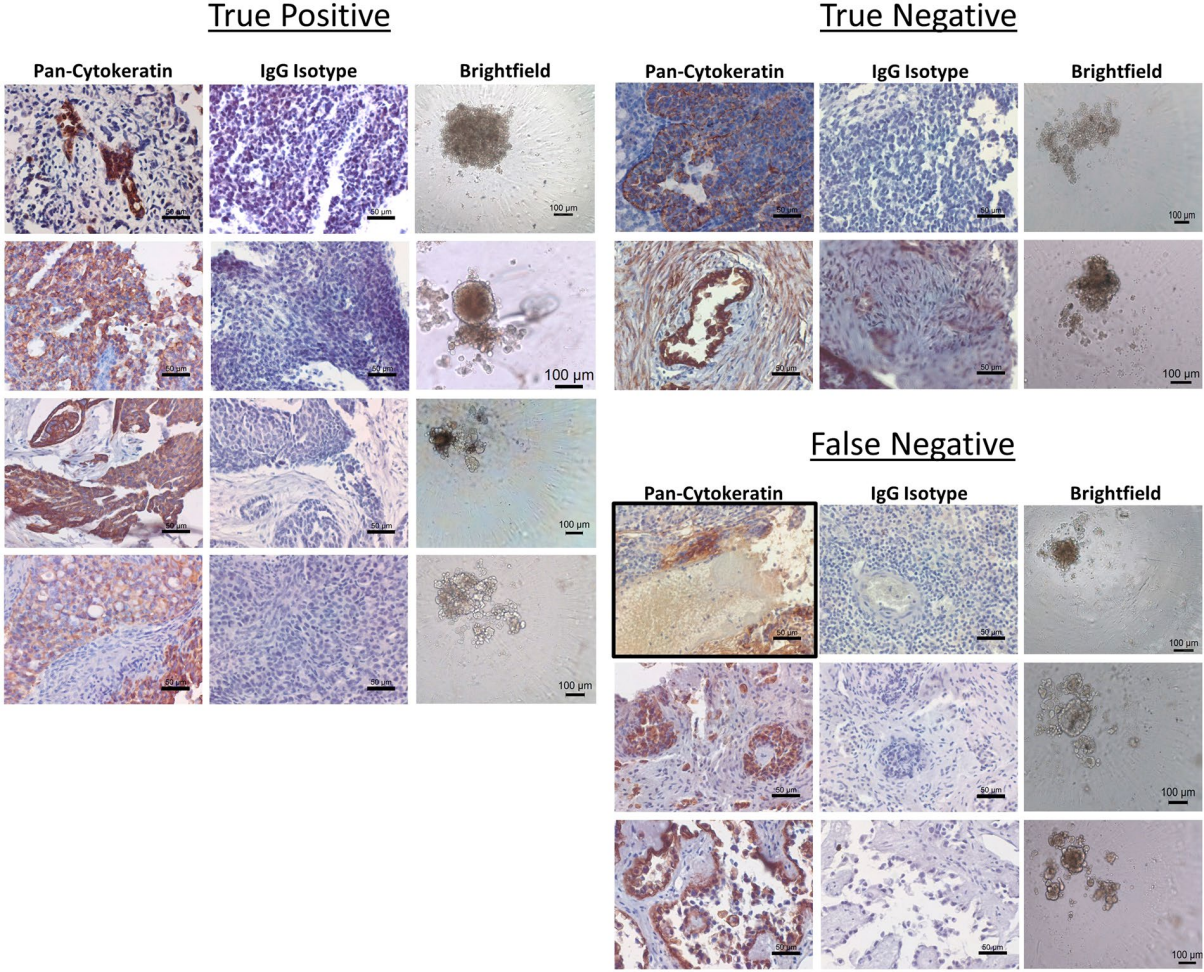
Representative imaging of primary debulking tissue samples and 3D spheroids formed for assay performance. Formalin fixed, paraffin embedded sections were stained for pan-cytokeratin expression and isotype control to identify the presence of epithelial cells in the tumor tissue, scale bars = 50 microns. Following assay performance, images were taken of representative 3D spheroids in the untreated control groups to verify the formation of spheroids during assay performance, scale bars = 100 microns.

In order to determine the accuracy of test predictions, we compared the test response category of the tested tissues to the clinical response of the enrolled patient on an individual level (Fig. 3). When determining if a patient was a Responder or Non-Responder to clinical combination therapy of carboplatin and paclitaxel, they were deemed to be a Responder if their test response fell within the Responder category for either carboplatin or paclitaxel. If their test response prediction indicated non-response to carboplatin and moderate or non-response to paclitaxel, then they were considered a Non-Responder. For two patients, OVC13 and OVA71, doxorubicin and gemcitabine were added to their initial 6 cycles of chemotherapy respectively. OVC13 had no test response to carboplatin or paclitaxel but did have test response to doxorubicin and was therefore classified as a true positive. OVA71 had no test response to carboplatin, paclitaxel, or gemcitabine and was therefore classified as a true negative. Histology of the primary tumor samples received revealed pan-cytokeratin positive cells indicating the presence of epithelial cells and these samples were verified to be ovarian cancer through pathology. Importantly, all samples formed spheroids in the 3D cell culture system although the size, shape, and compaction of the spheroids differed from sample to sample (Fig. 4).

**Figure 5:**
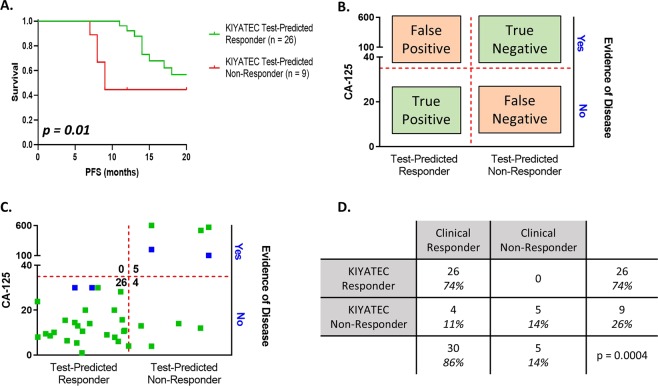
Assay response in newly diagnosed patients who underwent primary debulking surgery prior to platinum-based chemotherapy initiation. (**A**) Progression-free survival Kaplan-Meier plots of all patients within this cohort out to 20 months following initial surgery. The p value was calculated by the Wilcoxon test. (**B**) Defining parameters for categorizing patients as true positive, true negative, false positive, and false negative according to assay response and clinical response 6 months after the completion of first-line chemotherapy. Evidence of disease is defined by imaging analysis or physician notes. The horizontal red dashed line indicates a CA-125 value of 35 U/mL. The vertical red dashed line defines the break between assay Responders and assay Non-Responders. (**C**) Scatter plot of all patients analyzed within this population cohort. The green dots indicate clinical response defined by CA-125 with or without imaging, and the blue dots indicate clinical response defined only by imaging. (**D**) Contingency table of response cohorts. The p value was determined by Fisher’s exact test.

In addition to predictive accuracy we also examined the prognostic capability of the test in newly diagnosed ovarian cancer patients that receive upfront primary debulking followed by adjuvant platinum-based chemotherapy (Fig. 3). A Kaplan-Meier survival curve (Fig. 5A) indicated a significant difference in median progression-free survival (PFS) from surgery for those patients that were prospectively test-predicted to respond to first-line platinum-based chemotherapy (greater than 20 months) compared to those that were test-predicted to not respond (nine months) (HR 0.3414, 95% CI 0.08927 to 1.305, p = 0.01). First-line chemotherapy took an average of five months to complete resulting in only about a four-month chemotherapy free period for the test-predicted Non-Responders.

Prospective test response was also interrogated for its ability to predict an individual patient’s platinum sensitivity based upon the clinical criteria of PFS greater than six months following the completion of chemotherapy. For this analysis, test response was correlated to clinical response (CA-125 and radiographic assessment as interpreted by the physician) at least six months following the completion of first-line, platinum-based chemotherapy (Fig. 5B,C). Patients were classified into four categories based upon test response and prospective clinical assessment (Fig. 5B). True positives (TP) were defined as test-predicted Responders with no evidence of disease (NED) as assessed by radiographic imaging and/or a CA-125 < 35 U/mL at six months post-chemotherapy completion, and true negatives (TN) were defined as test-predicted Non-Responders with evidence of disease as assessed by radiographic imaging and/or a CA-125 value > 35 U/mL within six months post-chemotherapy prediction. Utilizing these three measurements, test response, radiographic assessment, and clinical CA-125, the predictive accuracy of the assay for platinum sensitivity was 31/35 (89%) (Fig. 5C,D).

The correlation of test predictive response with clinical response was statistically significant (p = 0.0004) as calculated by Fisher’s exact test with a test sensitivity of 87% (95% CI 70 to 94.7) and specificity of 100% (95% CI 56.6 to 100) (Fig. 5D). All 26 adjuvant patients identified as Responders by the test, TP, had no evidence of disease progression at least six months post-chemotherapy indicating clinical classification as “platinum sensitive”. Of the test-predicted Non-Responders (n = 9), three were refractory with progression within the first three months of treatment and two had clinical evidence of disease progression and relapse within six months of treatment completion as defined by an increase in tumor size and/or an increase in CA-125 and were successfully identified as resistant by the test assay, TN. Importantly, all of the five carboplatin/paclitaxel test-predicted Non-Responders had at least test-predicted Moderate Response to other drugs in the six drug panel, including doxorubicin and topotecan, suggesting they may have responded better clinically to a change in treatment. The final four patients were falsely identified by the test as Non-Responders but had a clinical response (false negative (FN)). There were zero false positives (FP).

### Test false negative response may be related to CA-125 values directly prior to surgery

**Figure 6:**
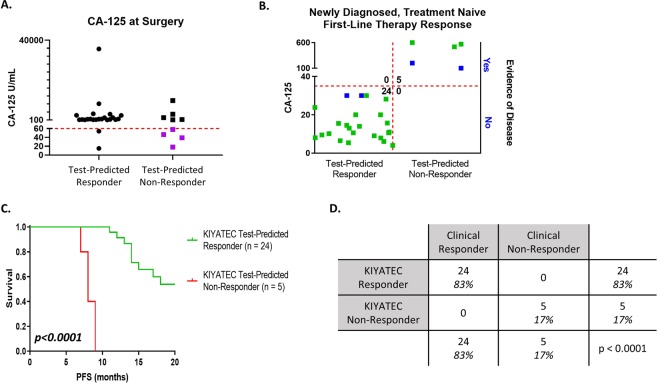
Upfront cutoff of patients with low CA-125 values at the time of surgery improves assay accuracy. (**A**) The CA-125 values for all patients in the newly diagnosed, primary debulking cohort were graphed by their assay response category. The purple dots were the 4 false negative readouts. The red dashed line indicates an initial CA-125 cutoff value of less than 60. (**B**) Scatter plot of all patients analyzed within this new population cohort. The green dots indicate clinical response defined by CA-125 with or without imaging, and the blue dots indicate clinical response defined only by imaging. (**C**) Progression-free survival Kaplan-Meier plots of all patients within this cohort out to 20 months following initial surgery. The p value was calculated by the Wilcoxon test. (**D**) Contingency table of response cohorts. The p value was determined by Fisher’s exact test.

In an effort to determine why the test was providing a limited number of FN readouts, we examined multiple factors, including grade, stage, and histology, none of which provided any indication of differentiation between groups. FN readouts could also be due to the tissue received for testing. However, only one sample, OVB27, indicated a low amount of pan-cytokeratin positive cells (Fig. 3, outlined in black). One difference that did standout was the distribution of CA-125 values at the time of surgery, prior to obtaining the tissue and completely unrelated to the test. The FN group had a mean CA-125 at surgery of 40.38 (Range 18 to 58) and the TN group had a mean CA-125 of 2895 (Range 151.4 to 9796). Importantly, this distinction was not present in a comparison of the TP (mean 2702, Range 15 to 35685) to the TN patients indicating that initial CA-125 immediately prior to surgery is not a good predictor of patient response to platinum-based chemotherapy. The initial CA-125 of the four FN patients was also significantly lower than the combined TN and TP groups of correctly predicted patients (group mean = 2737, FN mean = 40.4, p = 0.0474) (Fig. 6A). Clinically, patients with low CA-125 prior to surgery are known to have significantly superior prognosis^31–33^. When coupled with the discordant test results for these four patients, it is possible that the test’s prediction algorithm may be modified in the future for this small minority patient subpopulation. With this observation, we set a cutoff of 60 U/mL CA-125 as an exploratory disqualifier for the test and applied it to the entire 35 patient population of the primary debulking adjuvant group (Fig. 6A). This removed all four FN patients and two TP. The resulting data set is 100% accurate with 24 TP patients and five TN patients and no FP or FN patients (Fig. 6B,D). Importantly, while the diagnostic odds ratio with the FN readouts was 64.6, indicating a very good test, when they are removed, it increases to 544^34,35^. Additionally, PFS in the test-predicted Responders remained significantly different from PFS in the test-predicted Non-Responders with a median PFS of 21 months and eight months respectively (HR 0.0002, 95% CI 0.00001 to 0.003, p < 0.0001, Fig. 6C). We will continue to monitor the datasets as more patients are added to see if this exploratory cutoff ultimately plays a significant role in test accuracy.

### Test response predicts future clinical response to neoadjuvant chemotherapy in newly diagnosed ovarian cancer

**Figure 7:**
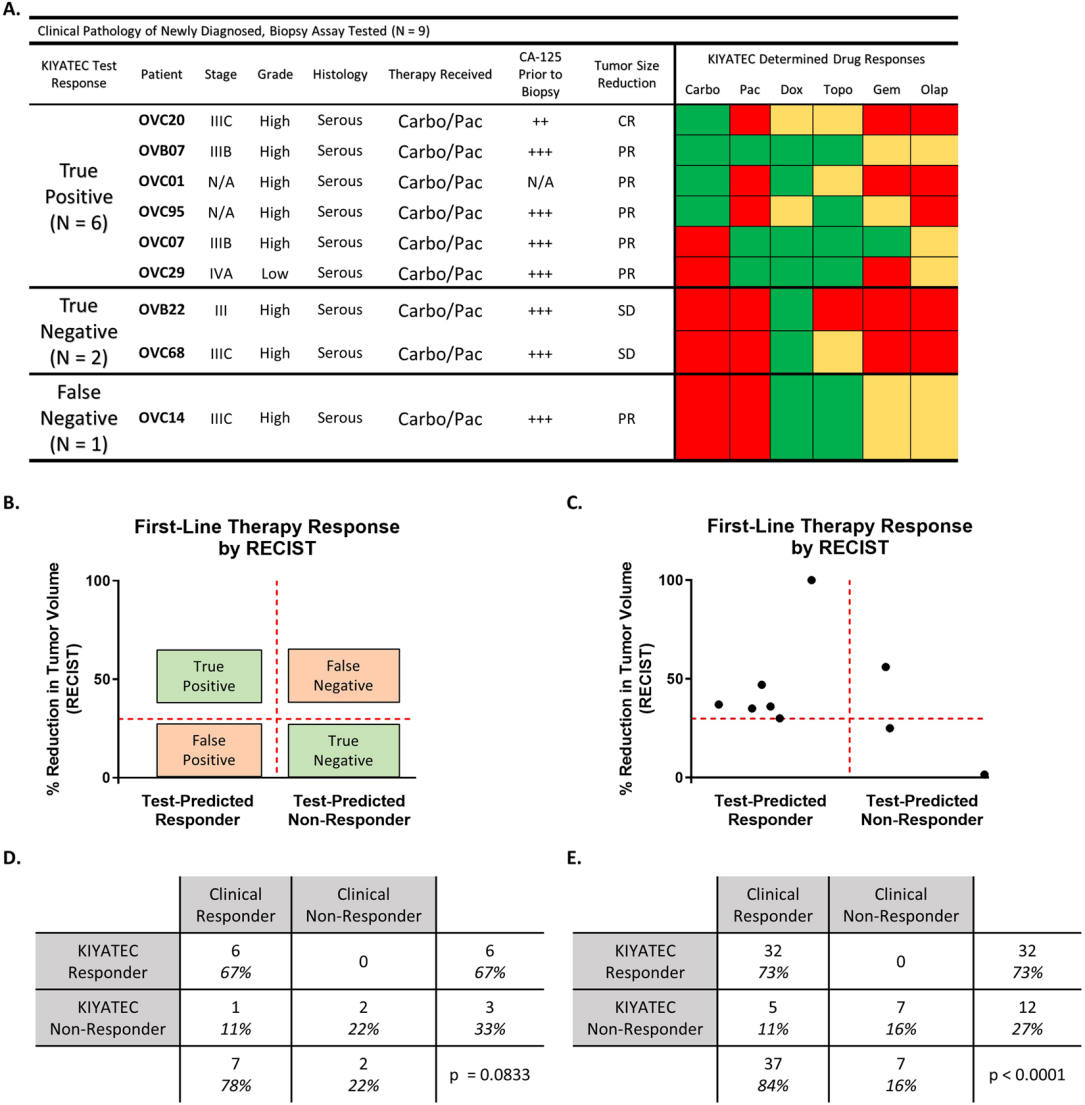
Assay response in newly diagnosed patients who underwent laparoscopic biopsy prior to initiation of neoadjuvant chemotherapy. (**A**) Clinical pathology of all patients in this group separated by their predicted response in the assay and in the clinic. Green indicates a Test Responder, Yellow a Test Moderate Responder, and Red a Test Non-Responder. Abbreviations: Carbo = carboplatin, Pac = paclitaxel, Dox = doxorubicin, Topo = topotecan, Gem = gemcitabine, Olap = olaparib; ++ = CA-125 > 60, <500, +++ = CA-125 ≥ 500; CR = complete response, PR = partial response, and SD = stable disease. (**B**) Defining parameters for categorizing patients as true positive, true negative, false positive, and false negative according to assay response and RECIST 1.1 criteria. The horizontal red dashed line indicates the 30% reduction in the sum of the target lesions that defines the minimum for defining partial response (PR). The vertical red dashed line defines the break between assay Responders and assay Non-Responders. (**C**) Scatter plot of all patients analyzed within this population cohort. (**D**) Contingency table of response cohorts for the laparoscopic biopsy group. (**E**) Contingency table of response cohorts for the combination of all newly diagnosed, treatment naïve ovarian cancer patients with assay response data and clinical response information. The p value was determined by Fisher’s exact test.

**Figure 8:**
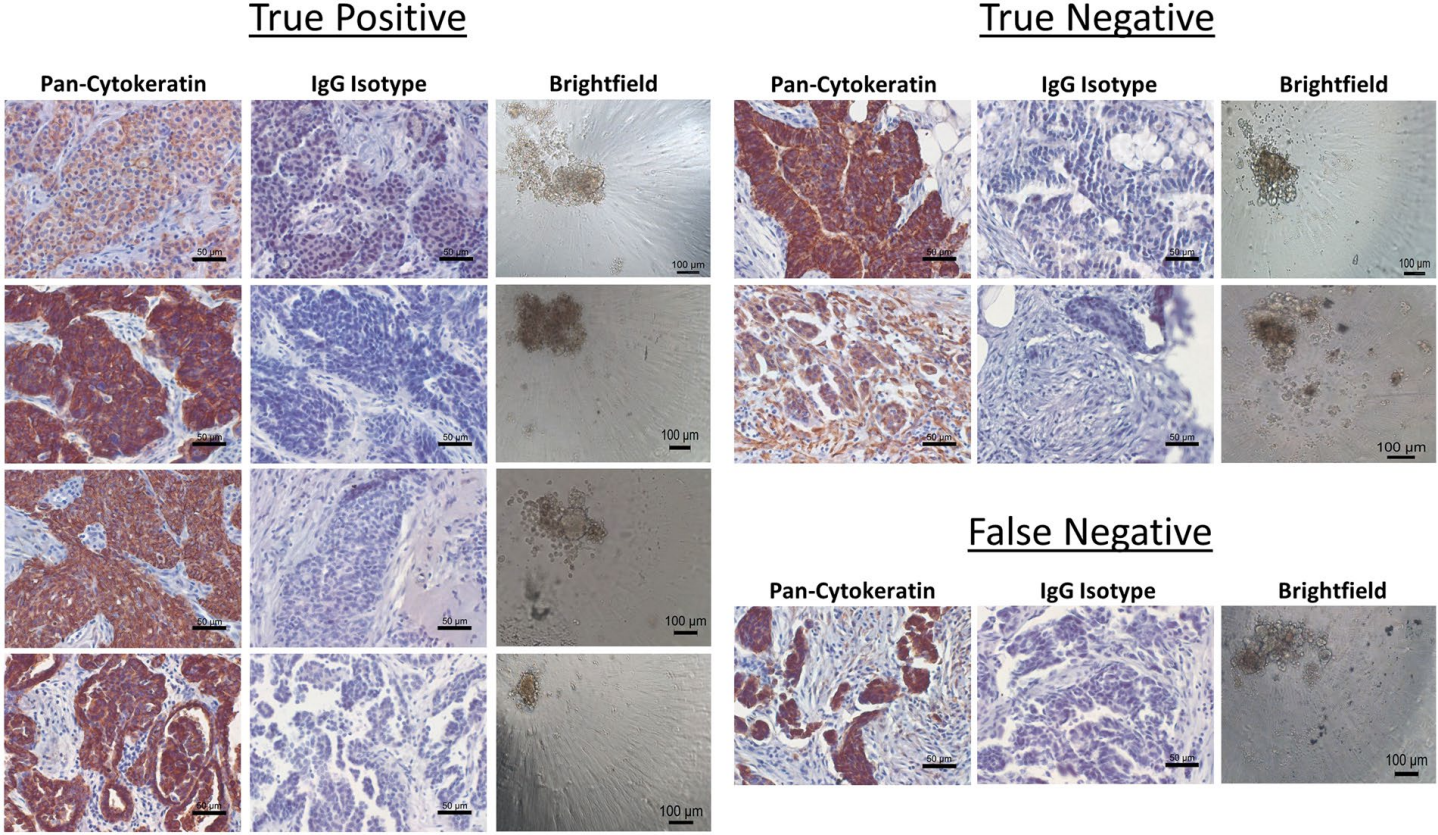
Representative imaging of laparoscopic biopsy tissue samples prior and 3D spheroids formed for assay performance. Formalin fixed, paraffin embedded sections were stained for pan-cytokeratin expression and IgG isotype control to identify the presence of epithelial cells in the tumor tissue, scale bars = 50 microns. Following assay performance, images were taken of representative 3D spheroids in the untreated control groups to verify the formation of spheroids during assay performance, scale bars = 100 microns.

We used the same test to predict response to neoadjuvant chemotherapy in patients who received a laparoscopic biopsy rather than an upfront debulking surgery (Fig. 7). In order to correlate test response to clinical response, we utilized the rules established by response evaluation criteria in solid tumors (RECIST 1.1) to classify the patients as complete response (CR), partial response (PR), progressive disease (PD), or stable disease (SD)^30^ (Fig. 7B). We then correlated test-predicted Response to CR and PR and test-predicted Non-Response to PD or SD. After examining nine patients, one was CR and correlated with test-predicted Response to carboplatin (TP), six were PR and five of those six correlated with test-predicted Response to carboplatin and/or paclitaxel (TP), and two were SD with correlation to test-predicted Non-Response to both carboplatin and paclitaxel (TN) (Fig. 7A,C). The single FN patient had PR but did not respond to carboplatin or paclitaxel in the test. This resulted in an accuracy in this population of 89% (8 of 9), p = 0.0833 (Fig. 7D), The sensitivity was 86% (95% CI 48.7 to 99.3) and the specificity was 100% (95% CI 17.8 to 100) which are similar to the results from the adjuvant treated newly diagnosed patients (Fig. 5D). Importantly, when predictions in newly diagnosed adjuvant treated ovarian cancer are combined with predictions in neoadjuvant treated ovarian cancer, an accuracy of 89%, p < 0.0001 is established (Fig. 7E). This results in an overall test sensitivity of 86% (95% CI 72 to 94) and test specificity of 100% (95% CI 64.6 to 100). We also examined the neoadjuvant samples we received for tumor cells by staining for pan-cytokeratin (Fig. 8). These samples also formed a variety of spheroid shapes and sizes. Taken together, this data indicates the potential to use this test in multiple settings in newly diagnosed patients for an accurate prediction of first-line therapy response in ovarian cancer.

## Discussion

No routinely used test accurately resolves an ovarian cancer patient population into future chemotherapy responders or non-responders prior to treatment initiation. Here we present data on the analytical validation of such a test and in a prospective, blinded clinical study we have established that the test is able to accurately do so 89% of the time. In a study population of 44 newly diagnosed ovarian cancer patients who received first-line, platinum-based chemotherapy, the 32 prospectively test predicted Responders had an eventual clinical response rate of 100%. The test had the same level of correlative response prediction accuracy for both adjuvant and neoadjuvant treated groups. Within this group of patients this knowledge may have particular benefit for older patients and/or those with significant co-morbidities for whom toxicity of a prospective chemotherapeutic is weighed heavily in decisions to treat. Test data were generated within seven days of debulking surgery or laparoscopic biopsy, a clinically relevant timeframe relative to when treatment decisions are made and communicated.

Despite a lack of controls for grade, stage, or success of surgery, the 12 prospectively test-predicted Non-Responders had a correspondingly poor clinical response rate of 42% (5 of 12). The low clinical response rate of the test-predicted Non-Responders establishes a comparative metric against which to weigh alternative treatment plans with a response rate higher than 42%. Further, we determined that the false negatives in the adjuvant treated group had a relatively low pre-operative CA-125 (<60 U/mL) compared to most of the population (29 of 35) with a CA-125 greater than 100 U/mL. In this elevated CA-125 majority population of 29 patients, the test-predicted Non-Responders were associated with a clinical response rate of 0%. At this level of test accuracy, any alternative treatment strategy with a non-zero predicted response rate could be considered and prioritized. With planned additional clinical validation in a larger study, the test is positioned to enable a strategy to raise the current 70–80% response rate even higher in newly diagnosed ovarian cancer and potentially increase the current poor overall survival.

Differentiation between this test and previous chemoresponse assays is evident via direct comparison of techniques. This test’s superior performance may, in part, be attributable to the differences between the culture conditions, i.e., the advantages of a 3D microenvironment over 2D cell culture. In representative previous chemoresponse testing^36,37^, a 2D assay was only successfully completed on 78% of eligible enrolled patients compared to the current 90% success rate of the test demonstrated in this paper. Additionally, the previous tests generally required longer time in culture, increasing not only turnaround time but also the amount of molecular changes that typically occur in the cells due to microenvironmental insults from 2D cell culture conditions^38,39^. Because of the platform and performance challenges of using 2D cell culture-based tests to accurately predict response, alternative 3D platforms have been explored including organoid cultures^40^. While these have been successful in terms of clinical predictivity, these organoid culture systems can take six to eight weeks to generate a response prediction, limiting their clinical utility for most patients’ drug selection needs. This further highlights the strength of the seven-day turnaround time for the test validated in this study, which maximizes clinical utility and in general is quicker than the turnaround time of many other tests on which drugs are suggested. However, due to the reliance of the test validated in this study upon live cells, test utilization is limited to those tumor types from which sufficient tissue and live cells can be obtained such as ovarian cancer or glioblastoma in which surgical resection is the standard of care. To combat this issue, studies are currently being performed to analytically validate techniques to utilize the test from a single diagnostic biopsy core. There techniques will then be applied to tumor types such as breast cancer and others that rely heavily upon neoadjuvant therapy prior to debulking surgery or relapse situations where full debulking surgery is not warranted or feasible.

In summary, this study provides analytical validation and the initial prospective clinical validation of the use of a test to generate patient-specific evidence of response to first-line chemotherapy in newly diagnosed ovarian cancer patients prior to treatment initiation. The test data has a very high correlation to patient-matched clinical outcomes of corresponding response or non-response and encompasses drugs received by the patients as part of their first-line and/or second-line therapy. The high correlation against prospective clinical response (p < 0.0001), seven-day turnaround, and ability to predict both effective and ineffective treatments provides preliminary clinical demonstration of the validated test as a powerful tool in personalized cancer care. When validated in larger, independent cohorts, this test represents a new class of test designed to support clinical treatment decisions in oncology with strong clinical utility.

## Methods

### Cell lines and reagents

Ovarian cancer cell line A2780 was purchased from Sigma (St. Louis, MO). OVCAR-3 cell line was purchased from ATCC (Manassas, VA) while OVCAR-8, SK-OV-3, OVCAR-5, and OVCAR-4 were procured from NCI (Bethesda, MD). All cell lines were cultured as suggested in RPMI-1640 supplemented with 10% Fetal Bovine Serum and 1% Penicillin/Streptomycin and all primary tissues were cultured in DMEM supplemented with 10% Fetal Bovine Serum and 1% Penicillin/Streptomycin. All media components were sourced from Mediatech (Manassas, VA). All cell lines were kept at 37 °C and 5% CO_2_ with media changes every other day and passaged once 90% confluency was reached. All drugs were sourced from SelleckChem (Houston, TX).

### Patients and study design

After providing written informed consent, patients >18 years of age with suspected or known ovarian cancer were enrolled onto a multi-site, prospective, non-intervention, Institutional Review Board (IRB) approved biology protocols by Prisma Health, formerly known as Greenville Health System, Cancer Institute (IRB-Committee C), Mayo Clinic (Mayo Clinic IRB), Saint Thomas/Ascension (Sterling IRB), and Inova Health System (Inova IRB). Tissues were also purchased from iSpecimen under their IRB protocols. Tissue acquisition was carried out in accordance with the guidelines and regulations specified by each institution and informed consent was obtained from all participants. Included in this analysis are patients with newly diagnosed, histologically confirmed high grade epithelial ovarian, fallopian tube, or primary peritoneal carcinoma. Patients received clinical care according to standard practice and results of the assay were not provided to the patient or physician. Clinical follow-up 12 months from time of enrollment and standard clinical endpoints (history, physical exams, CA-125, and radiographic assessments) were used to assess positive or negative clinical response to chemotherapy. This report focuses on newly diagnosed ovarian cancer patients receiving adjuvant chemotherapy after primary surgical debulking and patients receiving neoadjuvant therapy following laparoscopic biopsy.

### Histology

Upon receipt of fresh tissue, a piece was removed and immediately fixed in formalin. The fixed tissue was processed and embedded in paraffin. For histology staining, 5 µm sections were mounted onto glass slides. Rehydration and antigen retrieval were performed using citrate buffer, pH 6.0 (Abcam, Cambridge, UK). Slides were stained with Anti-pan Cytokeratin AE1/AE3 + 5D3 (Abcam, Cambridge, UK) or IgG Isotype control (Abcam, Cambridge, UK) for 2 hours at room temperature. Staining was detected using Mouse and Rabbit Specific HRP/DAB IHC Detection kit (Abcam, Cambridge, UK). Images were taken on an Olympus IX70 microscope with a Jenoptik (Jena, Germany) ProgRes C14plus camera and ProgRes CapturePro software. Brightfield spheroid images were taken on the same microscope with the same camera and software.

### Assay performance

Live, fresh tissue was collected at surgical debulking or laparoscopic biopsy of primary tumor sites. Within 24 hours of surgery, the tissue was received and immediately enzymatically dissociated into single cells. The cells were formed as spheroids for 24 hours in 384-well spheroid microplates (Corning Inc., Corning, NY) after which time they were exposed to standard of care chemotherapies as single agents for 72 hours. Drugs were dosed from 0.005 µM to 100 µM and response was measured via CellTiter-Glo 3D (Promega, Madison, WI). Cell viability was calculated for each concentration as an average of seven replicates and normalized to untreated vehicle controls. Additional controls included vehicle blanks and high and low positive kill controls. Thresholds of assay success were set to include minimum Relative Luminescence Units (RLU) values in controls and the dynamic range between vehicles and blanks. Results were obtained within 7 days of tissue receipt and maintained in a database while patients received standard treatment in the clinic. Technicians remained blinded to all clinical characteristics of the tissues during performance of the test other than that they were running the ovarian cancer test. Importantly, because the assay was performed immediately upon fresh tissue receipt, there were a small number of cases that were determined by pathology to be either benign or not ovarian in origin after the assay was performed. These cases were eliminated from all downstream analysis and their numbers are not included in any datasets in this report.

### Test response determination

For each drug tested, either a binary Response/Non-Response prediction or a ternary Response/Moderate Response/Non-Response prediction were established dependent upon the available clinical follow-up data. For carboplatin response determination, sufficient clinical data was available to establish binary Response/Non-Response predictions and confirm cutoffs through ROC curve analysis (AUC = 0.9630, 95% CI 0.8998 to 1.0 p = 0.0012). Without sufficient clinical data to establish such binary predictions, IC50 thresholds separating test-predicted Response and test-predicted Non-Response were determined experimentally for each drug tested using test response distributions informed by quartile analysis of test data from multiple patient samples. Using results from all patient samples tested, IC50 values were collated for each individual drug and cutoffs informed by the approximate 25^th^ and 75^th^ quartiles were used to determine predicted Responders (IC50s below the lower threshold), Moderate Responders (IC50s between the lower and upper thresholds), and Non-Responders (IC50s above the upper threshold). When IC50 values fell outside of the range of concentrations tested, they were considered Non-Responders if too high. No tested sample’s response fell below the tested drug curve.

### Clinical data analysis

Based on their test results, patients were categorized as either test Responders or Non-Responders to individual chemotherapeutics, including carboplatin, paclitaxel, doxorubicin, and gemcitabine. For a patient to be considered a test predicted Responder to first-line combination chemotherapy, test results had to indicate response to at least one of the drugs received by the patient in the clinic and tested in the lab. If the test results indicated no response to any of the drugs received in clinic, then the patient was classified as a test predicted Non-Responder. Clinical determination of non-response or response for adjuvant treated patients was defined as either progression within 6 months following the conclusion of chemotherapy or progression free status at 6 months or longer following the conclusion of chemotherapy, respectively. Positive clinical response to chemotherapy was defined by CA-125 below 35 U/mL^41^ and/or radiographic assessment^42^ of no evidence of disease or stable disease. Negative clinical response was defined as CA-125 above 35 U/mL and/or radiographic evidence of tumor growth/spread. In both cases, radiographic assessment held weight over CA-125, as the biomarker is known to be be unreliable in some cases^43,44^. For neoadjuvant treated patients, CT imaging was used to determine the sum of diameters of target lesions as defined by RECIST 1.1 prior to laparoscopic biopsy and directly prior to interval debulking surgery. RECIST 1.1 criteria was then used to define patients as having a complete response (CR), partial response (PR), progressive disease (PD), or stable disease (SD).

### Statistical analysis

Statistical calculations were performed using Graphpad Prism 8.0. IC50 values were calculated using nonlinear regression normalized to vehicle treated controls. Primary analysis of test data and clinical outcomes utilized a Fisher’s Exact test to measure the statistical significance of the data distribution. Kaplan-Meier curves and Wilcoxon tests were used to examine the statistical associations between the test results and PFS.

## Data Availability

The datasets generated and analyzed during the current study are available from the corresponding author on reasonable request.

## References

[1] McGuire WP (1996). Cyclophosphamide and cisplatin compared with paclitaxel and cisplatin in patients with stage III and stage IV ovarian cancer. N Engl J Med.

[2] Torre LA (2018). Ovarian cancer statistics, 2018. CA Cancer J Clin.

[3] Soyama H (2017). Factors favouring long-term survival following recurrence in ovarian cancer. Mol Clin Oncol.

[4] van der Burg ME (2002). Weekly cisplatin and daily oral etoposide is highly effective in platinum pretreated ovarian cancer. Br J Cancer.

[5] Sundar S, Wu J, Hillaby K, Yap J, Lilford R (2012). A systematic review evaluating the relationship between progression free survival and post progression survival in advanced ovarian cancer. Gynecol Oncol.

[6] Eisenhauer EA (2017). Real-world evidence in the treatment of ovarian cancer. Ann Oncol.

[7] Morgan RJ (2012). Ovarian cancer, version 3.2012. J Natl Compr Canc Netw.

[8] Morgan RJ (2013). Ovarian cancer, version 2.2013. J Natl Compr Canc Netw.

[9] Markman M (1991). Second-line platinum therapy in patients with ovarian cancer previously treated with cisplatin. J Clin Oncol.

[10] Eisenhauer EA, Vermorken JB, van Glabbeke M (1997). Predictors of response to subsequent chemotherapy in platinum pretreated ovarian cancer: a multivariate analysis of 704 patients [seecomments]. Ann Oncol.

[11] Tsimberidou AM (2014). Personalized medicine for patients with advanced cancer in the phase I program at MD Anderson: validation and landmark analyses. Clin Cancer Res.

[12] Mao H, Lebrun DG, Yang J, Zhu VF, Li M (2012). Deregulated signaling pathways in glioblastoma multiforme: molecular mechanisms and therapeutic targets. Cancer Invest.

[13] Bastien JI, McNeill KA, Fine HA (2015). Molecular characterizations of glioblastoma, targeted therapy, and clinical results to date. Cancer.

[14] de Melo Gagliato D, Jardim DL, Marchesi MS, Hortobagyi GN (2016). Mechanisms of resistance and sensitivity to anti-HER2 therapies in HER2+ breast cancer. Oncotarget.

[15] Albain KS (2010). Prognostic and predictive value of the 21-gene recurrence score assay in postmenopausal women with node-positive, oestrogen-receptor-positive breast cancer on chemotherapy: a retrospective analysis of a randomised trial. Lancet Oncol.

[16] Sparano Joseph A., Gray Robert J., Makower Della F., Pritchard Kathleen I., Albain Kathy S., Hayes Daniel F., Geyer Charles E., Dees Elizabeth C., Goetz Matthew P., Olson John A., Lively Tracy, Badve Sunil S., Saphner Thomas J., Wagner Lynne I., Whelan Timothy J., Ellis Matthew J., Paik Soonmyung, Wood William C., Ravdin Peter M., Keane Maccon M., Gomez Moreno Henry L., Reddy Pavan S., Goggins Timothy F., Mayer Ingrid A., Brufsky Adam M., Toppmeyer Deborah L., Kaklamani Virginia G., Berenberg Jeffrey L., Abrams Jeffrey, Sledge George W. (2018). Adjuvant Chemotherapy Guided by a 21-Gene Expression Assay in Breast Cancer. New England Journal of Medicine.

[17] Brand TC (2016). Patient-specific Meta-analysis of 2 Clinical Validation Studies to Predict Pathologic Outcomes in Prostate Cancer Using the 17-Gene Genomic Prostate Score. Urology.

[18] Gray RG (2011). Validation study of a quantitative multigene reverse transcriptase-polymerase chain reaction assay for assessment of recurrence risk in patients with stage II colon cancer. J Clin Oncol.

[19] Fruehauf JP (2002). *In vitro* assay-assisted treatment selection for women with breast or ovarian cancer. Endocr Relat Cancer.

[20] Tian C (2014). Evaluation of a chemoresponse assay as a predictive marker in the treatment of recurrent ovarian cancer: further analysis of a prospective study. Br J Cancer.

[21] Cree IA (2007). A prospective randomized controlled trial of tumour chemosensitivity assay directed chemotherapy versus physician’s choice in patients with recurrent platinum-resistant ovarian cancer. Anticancer Drugs.

[22] Black MM, Speer FD (1953). Effects of cancer chemotherapeutic agents on dehydrogenase activity of human cancer tissue *in vitro*. Am J Clin Pathol.

[23] Herzog TJ, Krivak TC, Fader AN, Coleman RL (2010). Chemosensitivity testing with ChemoFx and overall survival in primary ovarian cancer. Am J Obstet Gynecol.

[24] Burstein HJ (2011). American Society of Clinical Oncology clinical practice guideline update on the use of chemotherapy sensitivity and resistance assays. J Clin Oncol.

[25] Bhadriraju K, Chen CS (2002). Engineering cellular microenvironments to improve cell-based drug testing. Drug Discov Today.

[26] Lee GY, Kenny PA, Lee EH, Bissell MJ (2007). Three-dimensional culture models of normal and malignant breast epithelial cells. Nat Methods.

[27] Suidan RS (2014). A multicenter prospective trial evaluating the ability of preoperative computed tomography scan and serum CA-125 to predict suboptimal cytoreduction at primary debulking surgery for advanced ovarian, fallopian tube, and peritoneal cancer. Gynecol Oncol.

[28] Horowitz NS (2015). Does aggressive surgery improve outcomes? Interaction between preoperative disease burden and complex surgery in patients with advanced-stage ovarian cancer: an analysis of GOG 182. J Clin Oncol.

[29] Eisenhauer EA (2009). New response evaluation criteria in solid tumours: revised RECIST guideline (version 1.1). Eur J Cancer.

[30] Rustin GJ (2011). Definitions for response and progression in ovarian cancer clinical trials incorporating RECIST 1.1 and CA 125 agreed by the Gynecological Cancer Intergroup (GCIG). Int J Gynecol Cancer.

[31] Gupta D, Lammersfeld CA, Vashi PG, Braun DP (2010). Longitudinal monitoring of CA125 levels provides additional information about survival in ovarian cancer. J Ovarian Res.

[32] Gupta D, Lis CG (2009). Role of CA125 in predicting ovarian cancer survival - a review of the epidemiological literature. J Ovarian Res.

[33] Davidson NG, Khanna S, Kirwan PH, Bircumshaw D (1991). Prechemotherapy serum CA125 level as a predictor of survival outcome in epithelial carcinoma of the ovary. Clin Oncol (R Coll Radiol).

[34] Glas AS, Lijmer JG, Prins MH, Bonsel GJ, Bossuyt PM (2003). The diagnostic odds ratio: a single indicator of test performance. J Clin Epidemiol.

[35] Simundic AM (2009). Measures of Diagnostic Accuracy: Basic Definitions. EJIFCC.

[36] Rutherford T (2013). A prospective study evaluating the clinical relevance of a chemoresponse assay for treatment of patients with persistent or recurrent ovarian cancer. Gynecol Oncol.

[37] Grendys EC (2014). Overview of a chemoresponse assay in ovarian cancer. Clin Transl Oncol.

[38] Breslin S, O’Driscoll L (2016). The relevance of using 3D cell cultures, in addition to 2D monolayer cultures, when evaluating breast cancer drug sensitivity and resistance. Oncotarget.

[39] Lee JM (2013). A three-dimensional microenvironment alters protein expression and chemosensitivity of epithelial ovarian cancer cells *in vitro*. Lab Invest.

[40] Vlachogiannis G (2018). Patient-derived organoids model treatment response of metastatic gastrointestinal cancers. Science.

[41] Skaznik-Wikiel ME (2011). Possible use of CA-125 level normalization after the third chemotherapy cycle in deciding on chemotherapy regimen in patients with epithelial ovarian cancer: brief report. Int J Gynecol Cancer.

[42] Esselen KM (2016). Use of CA-125 Tests and Computed Tomographic Scans for Surveillance in Ovarian Cancer. JAMA Oncol.

[43] Bischof P, Galfetti MA, Seydoux J, von Hospenthal JU, Campana A (1992). Peripheral CA 125 levels in patients with uterine fibroids. Hum Reprod.

[44] He RH, Yao WM, Wu LY, Mao YY (2011). Highly elevated serum CA-125 levels in patients with non-malignant gynecological diseases. Arch Gynecol Obstet.

